# Empowering expectant mothers: the role of digital-integrated GPNC in enhancing childbirth safety and experience

**DOI:** 10.3389/fpubh.2026.1865625

**Published:** 2026-10-21

**Authors:** Mingju Xiang, Ying Hu, Hongjing Chen, Mingfang Zhou

**Affiliations:** 1 Maternity Center, The Third Affiliated Hospital of Chongqing Medical University (FangDa Hospital), Chongqing, China; 2 Department of Anesthesiology Operating Room, The Second Affiliated Hospital of Chongqing Medical University, Chongqing, China; 3 Chongqing City Management University, Chongqing, China; 4 Chongqing University Industrial Technology Research Institute, Chongqing, China; 5 Maternity Center, The Third Affiliated Hospital of Chongqing Medical University (Fangda Hospital), Chongqing, China

**Keywords:** centering pregnancy, digital-integrated, distance education, group prenatal care, pregnancy pressure

## Abstract

**Background:**

This study was carried out to assess the effect of the Digital-integrated Centering Pregnancy group prenatal care (GPNC) on the pregnancy pressure and delivery experience in the third trimester.

**Methods:**

After obtaining consent, pregnant women who wanted to participate in the Digital-integrated GPNC (8–12 mothers per group, every 1 week for 1 consecutive month) during late pregnancy were included as the intervention group (*n* = 52). The control group (*n* = 48) was matched with the intervention for the week of gestation and age. The usual routine prenatal care model was implemented in both groups of pregnant women during late pregnancy. A structured questionnaire was applied to collect data consisting of the Personal Information Form, Pregnancy Pressure Scale (PPS), Childbirth Expectation Questionnaire (CEQ), the Chinese version of the Childbirth Experience Questionnaire (CEQ-C), and childbirth records in the intervention groups and the control groups. Data were analyzed using nonparametric tests and chi-square analysis, with baseline-adjusted sensitivity analyses performed to account for potential baseline differences.

**Results:**

In the post-test, PPS total scores of pregnant women in the intervention and control groups were significantly different, 0.51 (0.28, 0.69) vs. 0.66 (0.40, 1.09) (*z* = −2.178, *p* = 0.029), respectively. In addition, the CEQ score differed between the two groups (*p* < 0.05), being higher in the intervention group. After delivery, the two groups of primiparas who delivered naturally were evaluated in CEQ-C. There was a significant difference in the score of “Perceived safety” between the two groups (*p* < 0.05), the intervention group scoring was higher. Meanwhile, the total labor process and the first labor process time of primipara in the intervention group were shorter than those in the control group (*p* < 0.05).

**Conclusion:**

Using the Digital-integrated GPNC to implement distance education for pregnant women was associated with reduced prenatal pressure and improved childbirth expectancy, and showed a positive association with delivery experience.

## 1 Introduction

Pregnancy and childbirth are important events and special stages in a woman’s life. While looking forward to the arrival of a new life, pregnant women also experience major physiological and psychological changes (1, 2). Various pressures brought by this stress can affect the health of mothers and infants during pregnancy and postpartum (3). According to psychological research, the pregnancy stress index ranks 12th among the 43 items of the Social Life Stress Scale, thus suggesting that pregnancy is a great source of stress for pregnant women (4). Pregnancy itself brings physical discomfort and anxiety, such as first-trimester vomiting and fatigue. Meanwhile, the transition to motherhood introduces additional social, economic, and familial pressures. These combined factors make pregnant women vulnerable to psychological and emotional fluctuations, ultimately leading to pregnancy-related stress. A previous study (5) has shown that pregnancy-related psychological stress is a risk factor for abortion. Pregnant women under excessive psychological pressure have a higher probability of premature delivery and low birth weight infants. At the same time, a pregnant woman’s psychological problems can substantially affect the delivery outcomes and even postpartum lactation. Negative emotions such as anxiety and depression during pregnancy caused by pregnancy stress have been associated with adverse emotions such as postpartum depression (6). The social psychological stress pregnant women deal with differs in different pregnancy periods. Generally, the degree of pregnancy stress, fear, and anxiety is the lowest at 22–26 weeks, showing an upward trend after the third trimester of pregnancy (7). Therefore, the third trimester is considered an ideal period for pregnancy stress intervention (8). Learning how to overcome and alleviate pregnancy pressure is one of the important contents of prenatal learning for pregnant women.

In recent years, society and families have been focusing ever more on mothers’ and infants’ physical and mental health during pregnancy and childbirth. Existing research (9) shows that taking positive and effective methods and measures can improve pregnant woman’s social support and childbirth self-efficacy, thus effectively reducing pregnancy pressure. Health education is not only an intervention that can help pregnant women establish healthy behavior but is also an effective way to improve their self-care ability. Healthcare providers worldwide place great emphasis on antenatal and perinatal care, with particular attention to pregnancy health education. Such education serves as a proactive strategy to prevent psychological and physiological complications during labor and the postpartum period, while also reducing pregnancy-related stress and lowering the rate of cesarean sections (10, 11). Prenatal education counseling provided by medical staff has been proven to reduce the adverse psychology of pregnant women and improve their confidence in delivery (10). At the same time, a good medical pregnancy relationship can also promote the recovery of postpartum mothers and the care of newborns (12).

In the 1990s, American midwife Sharon Schindler Rising (13) introduced a new model of prenatal care called Centering Pregnancy (CP). This model is group-based. It integrates three core components: pregnancy examinations, health education, and social support. Pregnant women of similar gestational age groups are divided into 8–12, and group meetings are regularly organized. Each group session is preceded by one-to-one clinical assessments conducted by midwives. Pregnant women are actively involved in performing simple anthropometric and physiological measurements, including weight, blood pressure, abdominal circumference, and fetal heart rate. The recorded parameters are subsequently transcribed into the prenatal health documentation through a collaborative manual process between the women and the attending midwives. A health education theme is set at each meeting to encourage pregnant women to actively participate in the group discussion. This model advocates empowering pregnant women and encouraging them to choose a healthy lifestyle, thus enhancing their self-efficacy through group education. At the same time, the professional support of medical personnel, peer support among pregnant women, and other social support of this model alleviate woman’s negative emotions, such as tension, fear, and anxiety during pregnancy and childbirth, enabling them to deal with pregnancy pressure in a more positive manner (14–17). The widespread use of smartphones and tablets has changed people’s learning habits and methods. Identifying an appropriate and effective online health education model is particularly important to improve pregnant women’s physical and mental health. The popularity and operability of the Digital-integrated technology network widen the health service form provided by medical personnel and overcomes the limitations of traditional health education modes limited by time and space, perfectly meeting the requirements for developing maternal health. In recent years, pregnant women globally have faced growing demands for flexible, accessible prenatal education amid evolving healthcare needs and lifestyle changes. To alleviate pregnancy-related stress from multifaceted stressors and address women’s requirements for evidence-based knowledge on pregnancy, childbirth, and parenting, digital platforms have been widely integrated into prenatal health education (18). Mobile applications, online courses, and innovative forms such as virtual reality and interactive webtoons have emerged as effective tools to enhance health literacy, improve care adherence, and support emotional wellbeing (19–21). Digital interventions can significantly reduce prenatal anxiety (22), and WeChat-based education is widely accepted in Chinese public hospitals (23). A quasi-experimental study similar to ours found online education improved exclusive breastfeeding rates, further supporting digital model effectiveness (24). The digitally integrated GPNC model proposed in this study, which synergistically combines synchronous online interaction with asynchronous community-based support, conceptually bridges the structural void between individual mHealth applications—offering high accessibility yet limited social support—and conventional face-to-face GPNC interventions, which afford strong social cohesion but lack temporal and spatial flexibility.

This study explored the intervention effect of the Digital-integrated GPNC on pregnancy pressure and childbirth experience among women in the late stages of pregnancy to provide the scientific basis for promoting scientific, practical, and convenient maternal health care mode free from time and space restraints.

The present study is grounded in two complementary theoretical frameworks. First, the Health Belief Model (HBM) (25) provides the foundation for understanding how group-based learning cultivates collective self-efficacy in health management. Within the Digital-integrated GPNC, systematic health education and peer interaction reinforce women’s confidence in their ability to adopt health-promoting behaviors, as observing peers with similar gestational experiences successfully managing pregnancy-related challenges serves as cues to action and social reinforcement, thereby enhancing participants’ perceived self-efficacy in navigating pregnancy-related challenges. Second, House’s Social Support Theory (26) elucidates the multidimensional support mechanisms operationalized through both synchronous (Tencent Meeting) and asynchronous (WeChat group) interactions. The synchronous online sessions facilitate informational and instrumental support through structured health education delivered by midwives, while the asynchronous WeChat community sustains emotional and appraisal support through continuous peer interaction and shared experiences. Together, these frameworks explain how the hybrid digital model resolves the dialectical trade-offs between accessibility and social presence, providing a theoretical basis for understanding the mechanisms through which the Digital-integrated GPNC exerts its beneficial effects on pregnancy stress, childbirth expectations, and delivery experience.

## 2 Methods

### 2.1 Study design and sample

This was a quasi-experimental study with a control group. The study population was recruited from the maternity center of the Third Affiliated Hospital of Chongqing Medical University, China. Pregnant women who volunteered to join the four-week Digital-integrated GPNC were selected as the intervention group and were matched with the control group for the gestational week and age. Two groups of pregnant women were managed according to the routine prenatal care model in the third trimester of pregnancy (antenatal visits, offline maternity classes, and midwife clinics) (Figure 1).

**Figure 1:**
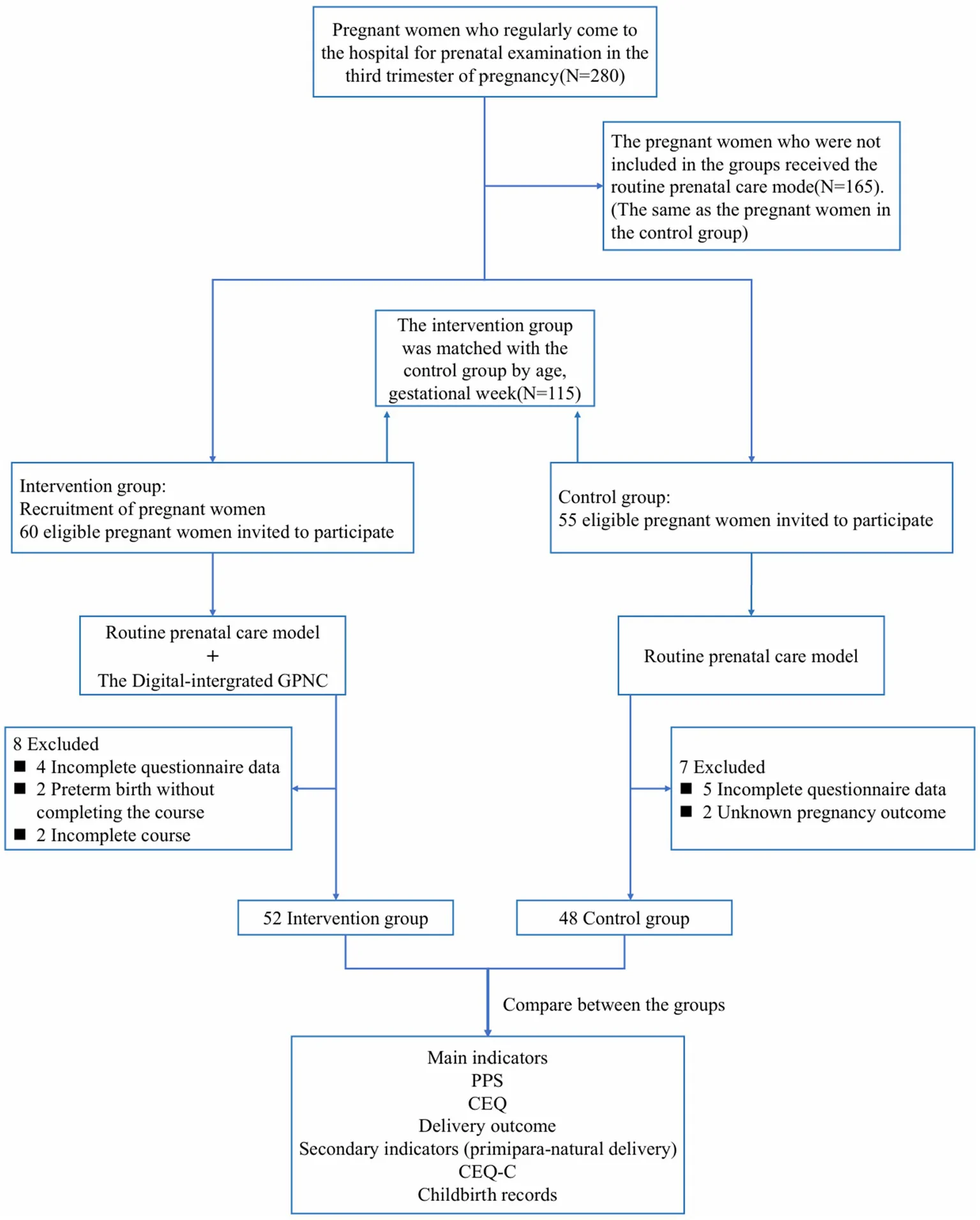
Patient enrollment and matching flow chart.

At the time of enrollment (T1), the subjects from the two groups scanned the App “questionnaire star” QR code on WeChat and filled in the Personal Information Form, PPS, and CEQ. After one month (four classes) of intervention (T2), they filled in the PPS and CEQ again during their regular prenatal examination at the hospital. Based on the data collected from two groups, we analyzed the intervention effect of the Digital-integrated Centering Pregnancy group prenatal care models. Within 3 days postpartum (T3), the researchers surveyed the primiparas from the two groups of natural delivery with the CEQ-C, collecting the data about their subjective feelings during delivery, and collected the data on the delivery situation of the primiparas from the two groups of natural delivery through the childbirth records, to understand the impact of the intervention scheme on the whole delivery experience. The filling time of each questionnaire was about 20–30 min (Figure 2).

**Figure 2:**
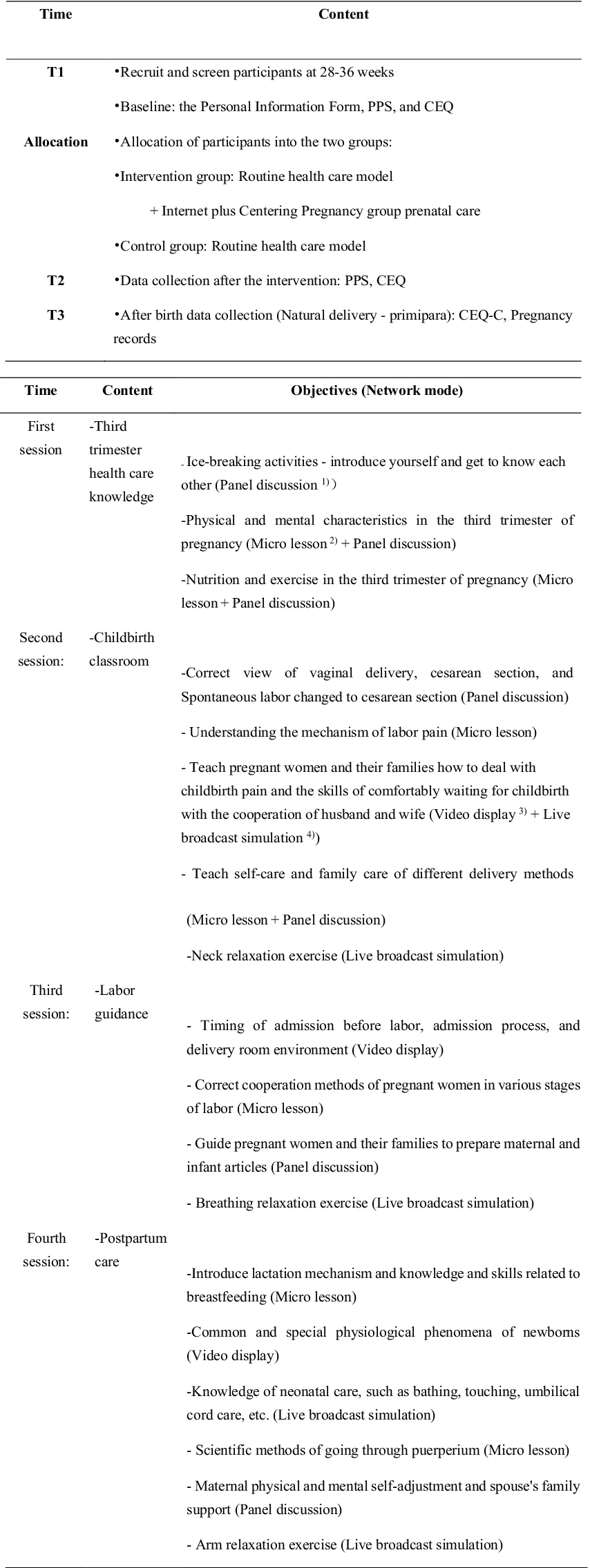
Key points for data collection. (1) *Panel discussion*: The medical staff established a WeChat group for the pregnant and childbearing family members, distributed the Tencent conference link website in the WeChat group in advance, opened the video function in the Tencent video conference, guided the group members to freely discuss the preset topics in the meeting, and finally made a summary of the discussion. (2) *Micro lesson*: Through the function of sharing the screen in the Tencent video conference, medical personnel explained the relevant knowledge related to pregnancy and childbirth that women in the third trimester and their families need to master in the form of slides. (3) *Video display*: Medical personnel used the function of sharing the screen in Tencent video conference to display the videos related to pregnancy and childbirth sorted out and recorded in advance. (4) *Live broadcast simulation*: Medical staff opened the video function in the Tencent video conference, conducted an online live broadcast, displayed the mother and child care operation, and evaluated the simulated operation of parents through the network.

### 2.2 Ethical considerations

In order to carry out this study, ethical approval was obtained from the ethics committee of the Third Affiliated Hospital of Chongqing Medical University (No. 68, 2023). Meanwhile, the study was prospectively registered in the China Clinical Trial Registration Center (Registration No.: ChiCTR2400089118) on January, 2024, prior to participant enrollment; All participants were fully aware of the objectives and process of the study, and written informed consent was obtained from all participants (pregnant women aged ≥18 years) prior to enrollment. They were also informed that the information obtained during the study would be kept confidential and that they could withdraw from the study at any time. The study was free of charge, and all the services were completely free.

### 2.3 Participants

In order to estimate the sample size, we used the variable mean score of pregnancy stress. In the pre-experimental phase, the mean score of PPS in late pregnancy was 0.70 ± 0.44. To detect effective between-group variation in pregnancy pressure (0.18 reduction in the mean score of pregnancy stress in the intervention group), with type I and type II errors of 5 and 10%, we had an effect size of 0.25. Assuming a dropout rate of 20%, a sample of 82 pregnant women was needed (41 in each group). Inclusion criteria were the following: ① pregnant women at 28–36 weeks of gestation and singleton head position; ② no abnormalities in B-ultrasound examination; ③ without mental and intellectual impairment, and with a certain understanding and language expression ability, who could independently complete the questionnaire; ④ pregnant women and their families informed consent and voluntarily participated in this study; Exclusion criteria were: ① abnormal obstetric conditions such as threatened preterm birth, premature rupture of membranes, cervical insufficiency or cervical cerclage; ② severe medical diseases such as preeclampsia or pregnancy-induced hypertension, pregnancy complicated with heart disease and pregnancy complications; ③ quit halfway or failed to complete the questionnaire. The intervention started in January 2024, and the follow-up ended in December 2025. Table 1 shows the access process of participants during the clinical trial.

**Table 1:** General characteristics of pregnant women in both groups.

Project	Intervention group (*n* = 52) frequency (%)	Control group (*n* = 48) frequency (%)	Test and *p*-value
Age			*x*^2^ = 3.501; *p* = 0.321
≤25	12 (23.1)	14 (29.2)	
26–30	29 (55.8)	18 (37.5)	
31–35	9 (17.3)	13 (27.1)	
≥36	2 (3.8)	3 (6.3)	
Degree of education			*x*^2^ = 4.805; *p* = 0.308
Junior high school and below	2 (3.8)	2 (4.2)	
High school or technical secondary school	6 (11.5)	9 (18.8)	
Junior college	14 (26.9)	19 (39.6)	
Undergraduate	26 (50.0)	14 (29.2)	
Master degree or above	4 (7.7)	4 (8.3)	
Occupation			*x*^2^ = 6.414; *p* = 0.378
Worker	5 (9.6)	5 (10.4)	
Personnel of enterprises and institutions	21 (40.4)	14 (29.2)	
Civil servant	2 (3.8)	0 (0.0)	
Individual operator	1 (1.9)	3 (6.3)	
Liberal professions	1 (1.9)	4 (8.3)	
None/unemployed	10 (19.2)	12 (25.0)	
Other	12 (23.1)	10 (20.8)	
Monthly disposable income			*x*^2^ = 2.705; *p* = 0.439
≤3,000	13 (25.0)	17 (35.4)	
3,001–5,000	27 (51.9)	25 (52.1)	
5,001–10,000	11 (21.2)	5 (10.4)	
≥10,001	1 (1.9)	1 (2.1)	
Childbirth payment method			*x*^2^ = 2.815; *p* = 0.421
Employee medical insurance	32 (61.5)	25 (52.1)	
Resident medical insurance	6 (11.5)	11 (22.9)	
The new rural coadjutant medical care	4 (7.7)	5 (10.4)	
At one’s own expense	10 (19.2)	7 (14.6)	
Marital relationship during pregnancy			*x*^2^ = 1.442; *p* = 0.230
Harmonious	44 (84.6)	36 (75.0)	
Occasional disagreement	8 (15.4)	12 (25.0)	
Relationship between mother-in-law and daughter-in-law			*x*^2^ = 4.781; *p* = 0.189
No mother-in-law	1 (1.9)	1 (2.1)	
Often quarrel	2 (3.8)	6 (12.5)	
Not much contact	36 (69.2)	24 (50.0)	
Very close	13 (25.0)	17 (35.4)	

*x*^2^: Pearson’s Chi-Squared Test.

### 2.4 Intervention

The Digital-integrated GPNC applied in the intervention group was based on the overseas centering pregnancy GPNC and completed online pregnancy education mode in line with China’s national conditions through the expert consultation method. The Digital-integrated GPNC in late pregnancy consisted of 3 modules, i.e., self-assessment of pregnant women to establish a pregnancy health file, Internet health education to complete online thematic education, and group online interaction to obtain social support. Each network course was headed by 1 senior midwife and 1 member of medical personnel. The pregnant women and their families were encouraged to attend courses simultaneously. The intervention process of this mode was as follows: (1) before the intervention, the pregnant women were contacted in advance through WeChat, forming groups of 8–12 groups of participants; (2) pregnant women with similar gestational weeks and their spouses were included in a WeChat group. The WeChat group was used for group members to discuss, interact and communicate relevant matters. The day before each class, the medical staff released the class information to the group through WeChat to remind pregnant women and their families not to miss the class; (3) Pregnant women and their families were guided on how to download the “Tencent Conference” App software in advance to facilitate the development of online education; the spouses were also required to participate, as there were many issues to be discussed and studied by couples, requiring interactive simulation. Husband’s support increased the spouse’s participation in the pregnant women’s pregnancy process. After each class, the maternity family theme education practice homework was recorded and presented as a video. The instructor controlled the practice of team members through the recorded family video, and each couple shared the practice experience before the next class; (4) Online education was arranged and implemented according to the curriculum (Table 2), which took the form of live broadcasts and interactive communication. It was held once a week on Saturday night, with 1.5 h per class each time, 4 classes per 4 weeks, totaling 6 class hours.

**Table 2:** Obstetric characteristics of pregnant women in both groups.

Project	Intervention group (*n* = 52) frequency (%)	Control group (*n* = 48) frequency (%)	Test and *p*-value
Gestational week			*x*^2^ = 0.875; *p* = 0.349
28–32	18 (34.6)	21 (43.8)	
33–36	34 (65.4)	27 (56.2)	
Birth experience			*x*^2^ = 2.541; *p* = 0.281
Primipara	43 (82.7)	36 (75.0)	
Previous natural delivery	7 (13.5)	6 (12.5)	
Previous cesarean section	2 (3.8)	6 (12.5)	
Self-health evaluation			*x*^2^ = 0.495; *p* = 0.482
Good	37 (71.2)	31 (64.6)	
General feeling	15 (28.8)	17 (35.4)	
Pregnancy plan			*x*^2^ = 0.083; *p* = 0.774
Yes	35 (67.3)	31 (64.6)	
No	17 (32.7)	17 (35.4)	
Influence of relatives and friends on delivery mode			*x*^2^ = 1.904; *p* = 0.168
Yes	4 (7.7)	8 (16.7)	
No	48 (92.3)	40 (83.3)	
Expected mode of birth			*x*^2^ = 2.410; *p* = 0.300
Natural childbirth	47 (90.4)	42 (87.5)	
Cesarean section	2 (3.8)	5 (10.4)	
Never thought	3 (5.8)	1 (2.1)P	

*x*^2^: Pearson’s Chi-Squared Test.

The network platform was used to present three intervention measures: ① self-test during pregnancy and establishment of online health files: pregnant women were guided to monitor their weight and fetal movement at home and record them in the daily online health files, to facilitate the remote monitoring of pregnant woman’s status by medical personnel. ② Online theme education: a fixed awareness and education theme was set each week, and online theme education was provided to pregnant families by displaying lessons on the theme of education. A pre-recorded video of the operation was shown, or the mold was used in a “Tencent meeting” for demonstration and explanation. At the same time, the information on problems of pregnant women in the group were collected one day before each class. Pregnant women shared their own dilemmas or concerns or issues related to mother and child problems they urgently wanted to learn more about. Medical staff urged everyone to discuss their issues in class. ③ Group online interaction to obtain all-round social support: medical staff shared relevant pregnancy and childbirth knowledge and maternal and infant care technology in the WeChat group every day, and pregnant families learned independently regardless of time and place restrictions. Group members freely communicated online in the form of discussion and interaction in the WeChat group, shared their experiences, thoughts, feelings, or dilemmas, encouraged each other, and offered peer support. Medical staff also gave timely and reasonable answers to relevant consultation questions in the group and provided professional support. All 52 participants in the intervention group completed the four-session Digital-integrated GPNC program and provided post-intervention data (T2), yielding a 100% retention rate. No participants withdrew from the study during the intervention period.

### 2.5 Data collection tools

#### 2.5.1 Personal information form

After consulting relevant literature and experts in obstetrics and gynecology, nursing, and psychology, we discussed and designed a questionnaire with the research group. The questionnaire consists of general data (such as age, education level, work situation, monthly family income, delivery payment method, etc.) and obstetric data (such as gestational week, childbirth experience, prenatal education, selection of expected delivery mode, etc.).

#### 2.5.2 Pregnancy pressure scale (PPS)

The pregnancy pressure was measured by the Pregnancy Pressure Scale (PPS) compiled by Li Dan et al. (46). This scale applies to the evaluation of pregnancy stress for pregnant women. It includes a total of 30 items across 3 dimensions, including *Parental role identity* (15 items), *Worry about maternal and child health* (8 items), and *Body shape pressure* (4 items). There are also three items not included in the above three dimensions, which are considered as *other factors*. The scale adopts a 4-level scoring method (0 = no stress at all; 1 = mild stress; 2 = moderate stress; 3 = severe stress), where the total score is obtained as the sum of 30 items. The higher the score, the greater the pregnancy pressure of pregnant women. The total average and sub-factor average scores are the average scores of the items contained in the scale; 0, 0.001–1.000, 1.001–2.000 and 2.001–3.000 represent none, mild, moderate, and severe, respectively. The Cronbach’s α coefficient for PPS in this study was 0.949 (27).

#### 2.5.3 Childbirth expectation questionnaire (CEQ)

The CEQ was compiled by Gupton et al. (28) and after modification and debugging (29), the Chinese version of the delivery expectation scale was formed in 1991. The scale is mainly used to evaluate the level of maternal delivery expectation. It has 37 items and 1 open question, with a total of 5 dimensions, including *medical environment* (13 items), *control and participation* (9 items), *spouse support* (6 items), *medical support* (4 items), and *labor pain expectation* (5 items). From 1 (totally disagree) to 5 (totally agree), a 5-level scoring method was adopted, in which the expected dimension of labor pain was inversely scored. The higher the score, the higher the expectation of delivery. The reliability coefficient of the scale was 0.86, and the content validity coefficient was 0.950.

#### 2.5.4 Chinese version of the childbirth experience questionnaire (CEQ-C)

In 2019, the adapted CEQ-C was compiled by Zhu et al. (30, 31). This scale is mainly used to evaluate the delivery experience of primipara in natural delivery. It contains 19 Likert items with scores ranging from 1 (totally agree) to 4 (totally disagree) points that measure the four main dimensions of the childbirth experience, i.e., *professional support* (6 items), *participation* (3 items), *own capacity* (6 items) and *perceived safety* (4 items). The dimensional score is the sum of the item codes in each dimension divided by the number of items. The total score is the sum of all item codes divided by the number of all items. The scores for the scale and every dimension range from 1 to 4 points, where higher ratings reflect more positive experiences. Cronbach’s alpha of the CEQ-C was 0.881.

#### 2.5.5 Childbirth records

The records form determines the observation index items of delivery by consulting relevant literature, which is used to collect the delivery conditions of primipara in two groups of natural delivery and carry out recruitment and sorting one by one according to the code of the research object, including the time of labor process, the amount of postpartum hemorrhage, neonatal Apgar score, lactation, etc.

## 3 Statistical analysis

SPSS 29.0 was used for data analysis and processing, and the Kolmogorov–Smirnov test was used for evaluation and normality testing. The frequency, percentage, median, and interquartile spacing were used to statistically describe the personal information of pregnant women and related scales. In addition, two independent sample *t*-tests or approximate *t*-tests and a chi-square test were used to determine the between-group differences. For non-normally distributed data, the nonparametric test method was used. A value of *p* < 0.01 or *p* < 0.05 represented statistically significant differences. Given the non-randomized design, we additionally performed baseline-adjusted sensitivity analyses to account for potential baseline differences between groups. Baseline comparability was verified across all outcome variables (all *p* > 0.05; Tables 3–6). Within-group pre-post comparisons were conducted using the Wilcoxon signed-rank test to control for individual baseline variation. Although Analysis of Covariance (ANCOVA) with baseline scores as covariates is the preferred approach for non-randomized pre-post designs, the non-normal distribution of our data (Kolmogorov–Smirnov *p* < 0.05 for most outcome variables) precluded its valid application. This limitation is acknowledged in the Discussion section.

**Table 3:** Between-group comparison of pregnancy stress levels of pregnant women in the third trimester before and after the intervention.

Project (score range)	Before intervention median (IQR)	After intervention median (IQR)	*z*	*p*
PPS total (0–3)				
Intervention group (*n* = 52)	0.56 (0.36–0.92)	0.51 (0.28–0.69)	−4.063	0.000^**α^
Control group (*n* = 48)	0.48 (0.30–0.83)	0.66 (0.40–1.09)	−1.297	0.195
*z*	−1.119	−2.178		
*p*	0.263*^b^*	0.029^*b^		
Parental role identity (0–3)				
Intervention group (*n* = 52)	0.33 (0.15–0.73)	0.30 (0.20–0.51)	−1.536	0.125*^α^*
Control group (*n* = 48)	0.23 (0.06–0.60)	0.40 (0.20–0.73)	−1.322	0.186
*z*	−1.541	−1.249		
*p*	0.123*^b^*	0.212*^b^*		
Worry about maternal and child health (0–3)				
Intervention group (*n* = 52)	1.00 (0.53–1.37)	0.75 (0.37–1.00)	−4.358	0.000^**α^
Control group (*n* = 48)	0.81 (0.53–1.21)	1.06 (0.40–1.46)	−0.719	0.472*^α^*
*z*	−1.184	−2.022		
*p*	0.237*^b^*	0.043^*b^		
Body shape pressure (0–3)				
Intervention group (*n* = 52)	0.75 (0.25–1.50)	0.75 (0.25–1.00)	−3.478	0.001^*α^
Control group (*n* = 48)	0.75 (0.00–1.25)	1.00 (0.25–1.87)	−0.232	0.816*^α^*
*z*	−0.186	−2.058		
*p*	0.853*^b^*	0.040^*b^		
Other factors (0–3)				
Intervention group (*n* = 52)	0.83 (0.33–1.33)	0.66 (0.33–1.00)	−1.964	0.050*^α^*
Control group (*n* = 48)	0.66 (0.33–1.00)	1.00 (0.33–1.33)	−1.541	0.123*^α^*
*z*	−1.299	−1.453		
*p*	0.194*^b^*	0.146*^b^*		

^α^Wilcoxon signed-rank test.

^b^Wilcoxon rank sum test.

^*^p < 0.05; ^**^p < 0.001.

**Table 4:** Between-group comparisons of delivery expectations of pregnant women in the third trimester before and after the intervention.

Project (Score range)	Before intervention median (IQR)	After intervention median (IQR)	*z*	*p*
CEQ total (1–5)				
Intervention group (*n* = 52)	4.28 (3.89–4.45)	4.32 (4.16–4.45)	−1.862	0.063*^α^*
Control group (*n* = 48)	4.18 (3.81–4.37)	4.25 (3.82–4.40)	−0.313	0.754*^α^*
*z*	−1.374	−2.210		
*p*	0.169*^b^*	0.027^*b^		
Medical environment (1–5)				
Intervention group (*n* = 52)	4.84 (4.48–5.00)	4.84 (4.61–5.00)	−1.226	0.220*^α^*
Control group (*n* = 48)	4.80 (4.48–4.92)	4.65 (4.30–4.90)	−0.577	0.564*^α^*
*z*	−0.870	−2.294		
*p*	0.384*^b^*	0.022^*b^		
Spouse support (1–5)				
Intervention group (*n* = 52)	4.41 (4.16–5.00)	4.83 (4.66–5.00)	−4.899	0.000^**α^
Control group (*n* = 48)	4.50 (4.00–5.00)	4.8 (4.00–5.00)	−0.866	0.387*^α^*
*z*	−0.039	−2.054		
*p*	0.969*^b^*	0.040^*b^		
Control and participation (1–5)				
Intervention group (*n* = 52)	4.55 (4.00–5.00)	4.77 (4.36–5.00)	−2.916	0.004^*α^
Control group (*n* = 48)	4.33 (3.91–4.77)	4.55 (4.00–5.00)	−0.482	0.630^α^
*z*	−1.104	−2.108		
*p*	0.270*^b^*	0.035^*b^		
Labor pain expectation (1–5)				
Intervention group (*n* = 52)	2.00 (1.20–2.75)	1.50 (1.05–1.80)	−3.867	0.000^**α^
Control group (*n* = 48)	1.80 (1.20–2.00)	1.40 (1.00–2.15)	−0.383	0.702*^α^*
*z*	−1.446	−0.921		
*p*	0.148*^b^*	0.357 *^b^*		
Medical support (1–5)				
Intervention group (*n* = 52)	4.50 (4.00–5.00)	4.75 (4.25–5.00)	−2.392	0.017^*α^
Control group (*n* = 48)	4.25 (4.00–5.00)	4.50 (4.06–5.00)	−0.780	0.436^α^
*z*	−0.791	−1.377		
*p*	0.429*^b^*	0.168*^b^*		

^α^Wilcoxon signed-rank test.

^b^Wilcoxon rank sum test.

^*^p < 0.05; ^**^p < 0.001.

**Table 5:** Comparison of delivery experience between the two groups of primiparas with natural delivery.

Project (score range)	Intervention group (*n* = 29) median (IQR)	Control group (*n* = 22) median (IQR)	*z*	p^*^
CEQ-C total	1.78 (1.65–2.00)	1.71 (1.55–2.05)	−0.391	0.696
Professional support	1.16 (1.00–1.58)	1.25 (1.00–2.00)	−0.845	0.398
Perceived safety	2.50 (2.00–2.87)	2.00 (1.50–2.50)	−2.549	0.011
Participation	2.00 (1.33–2.00)	1.83 (1.33–2.08)	−0.206	0.837
Own capacity	1.83 (1.33–2.16)	1.91 (1.62–2.33)	−0.834	0.405

^*^The Wilcoxon rank-sum test.

^α^Wilcoxon rank sum test.

**Table 6:** Comparison between the two groups of primiparas of childbirth records.

Project^*^	Intervention group (*n* = 29)	Control group (*n* = 22)	*z/x^2^*	*p*
Length of stay	4.00 (3.00–4.25)	4.00 (3.00–5.00)	−0.667	0.505*^α^*
Analgesia			2.756	0.097*^b^*
Epidural	13 (44.8)	15 (68.2)		
Local anesthesia/block	16 (55.2)	7 (31.8)		
Perineal condition			0.318	0.858*^b^*
Lateral incision	10 (34.5)	6 (27.3)		
Laceration	18 (62.1)	15 (68.2)		
Nothing	1 (3.4)	1 (4.5)		
Duration of labor process				
Total stage of labor	350.00 (272.50–615.75)	542.50 (336.25–726.25)	−2.026	0.043*^α^*
First stage of labor	300.00 (222.50–580.00)	450.00 (288.75–660.00)	−2.256	0.024*^α^*
Second stage of labor	37.00 (26.75–77.50)	55.50 (38.25–71.25)	−0.495	0.621*^α^*
Third stage of labor	7.00 (4.00–9.25)	5.00 (3.75–6.00)	−1.784	0.074*^α^*
Postpartum hemorrhage	250.00 (200.00–290.00)	225.00 (197.50–280.00)	−1.069	0.285*^α^*

^b^Pearson’s Chi-Squared Test.

^*^Natural delivery-primipara.

## 4 Results

### 4.1 Comparison of descriptive characteristics between the two groups of pregnant women

The general characteristics of pregnant women in the intervention group and the control group were compared using Pearson’s chi-squared tests, revealing no significant differences between the two groups (all *p* > 0.05; e.g., age χ^2^(3) = 3.501, *p* = 0.321; education χ^2^(4) = 4.805, *p* = 0.308; Table 1). Also, no statistically significant differences were found between the two groups in terms of obstetric characteristics (all *p* > 0.05; e.g., gestational week χ^2^(1) = 0.875, *p* = 0.349; birth experience χ^2^(2) = 2.541, *p* = 0.281; Table 2).

### 4.2 Changes in prenatal psychological stress levels within and between groups

At baseline, both groups reported mild stress levels, with no significant between-group differences in total Prenatal Psychological Stress (PPS) scores or any subdimensions (e.g., total PPS *z* = −1.119, *p* = 0.263; all *p* > 0.05). Within-group analyses revealed that the intervention group exhibited a significant downward trend in total PPS (*z* = −4.063, *p* < 0.001), “Worry about maternal and child health” (*z* = −4.358, *p* < 0.001), “Body shape pressure” (*z* = −3.478, *p* = 0.001), and “other factors” (*z* = −1.964, *p* = 0.050), whereas the control group showed a slight, non-significant upward trend across all subscales (all *p* > 0.05; e.g., total PPS *z* = −1.297, *p* = 0.195). Between-group comparisons after the intervention further confirmed that the intervention group scored significantly lower in total PPS (*z* = −2.178, *p* = 0.029) and in the two most responsive dimensions “Worry about maternal and child health” (*z* = −2.022, *p* = 0.043) and “Body shape pressure” (*z* = −2.058, *p* = 0.040; Table 3), with mean reductions exceeding 15% from baseline in these domains indicating that emotional anxiety and body-image concerns were the primary drivers of overall stress reduction.

### 4.3 Changes in childbirth expectations within and between groups

Pre-intervention, no significant between-group differences were observed in total Childbirth Expectation Questionnaire (CEQ) scores or any subscales (e.g., total CEQ *z* = −1.374, *p* = 0.169; all *p* > 0.05). Within-group comparisons showed that the intervention group demonstrated a favorable directional shift post-intervention, with “spouse support” (*z* = −4.899, *p* < 0.001), “control and participation” (*z* = −2.916, *p* = 0.004), and “medical support” (*z* = −2.392, *p* = 0.017) increasing significantly, and “labor pain expectation” (*z* = −3.867, *p* < 0.001) decreasing significantly, while total CEQ showed a favorable but non-significant trend (*z* = −1.862, *p* = 0.063); the control group exhibited no notable changes in any scores (all *p* > 0.05). Between-group post-test analyses revealed that the intervention group significantly outperformed the control group in total CEQ (*z* = −2.210, *p* = 0.027) and the three dimensions of “medical environment” (*z* = −2.294, *p* = 0.022), “spouse support” (*z* = −2.054, *p* = 0.040), and “control and participation” (*z* = −2.108, *p* = 0.035; all *p* < 0.05, Table 4). Notably, “spouse support” and “control and participation” emerged as the dimensions with the largest improvement margins, suggesting that enhanced partner involvement and perceived autonomy were key mechanisms underlying the overall enhancement of childbirth expectations.

### 4.4 Comparison of childbirth experience among Primiparas with vaginal delivery

Postpartum assessment using the Chinese version of the Childbirth Experience Questionnaire (CEQ-C) demonstrated that the intervention group reported a significantly higher level of “perceived safety” compared with the control group (*z* = −2.549, *p* = 0.011; Table 5). This finding underscores that the intervention not only improved antenatal psychological parameters but also translated into a more positive subjective childbirth experience, particularly in terms of feeling secure during labor.

### 4.5 Comparison between the two groups of primiparas of childbirth records

Objective labor records showed that the intervention group had significantly shorter total duration of labor (*z* = −2.026, *p* = 0.043) and first-stage labor time (*z* = −2.256, *p* = 0.024) compared with the control group (Table 6). These results indicate that the beneficial effects of the intervention extended beyond psychological improvements to include measurable enhancements in labor efficiency, further supporting its clinical utility in optimizing both maternal experience and obstetric outcomes.

## 5 Discussion

The advent of the “Digital-integrated” era and the development of information technology have substantially affected medical care, making mobile medical interventions a new medical approach that has become increasingly popular and widely used (32). Some reports pointed out that Internet queries are important for pregnant women to obtain relevant information during pregnancy and childbirth. Pregnancy, fetal development, delivery, newborn health, and nutrition are the topics with the most frequent queries on the Internet (33). However, the science and effectiveness of network information are uneven, and their correctness often needs to be verified. A previous study (34) showed that when pregnant women absorb relevant knowledge about pregnancy and childbirth on the Internet, they are easily misled by wrong information, which may cause anxiety and fear, aggravating their stress levels during pregnancy (35). For women, pregnancy itself is a stressful life event. The physiological and psychological changes pregnant women undergo may have different degrees of maladjustment, resulting in psychological pressure. Especially with the passage of pregnancy time, the pressure during pregnancy increases linearly. The closer women are to delivery, the more obvious is their physiological and psychological stress response to issues such as delivery and childcare. Pregnancy is accompanied by multifaceted physiological, psychological, and social transitions, which have become increasingly complex in modern contexts (36). Contemporary lifestyle shifts—including heightened work–family role conflict, reduced in-person social support, financial strain, and pervasive exposure to unregulated online health information—have significantly elevated the psychological burden on pregnant women (37, 38). These changes leave them facing unprecedented levels of worry, uncertainty, and emotional distress throughout gestation, particularly in late pregnancy. This study evaluated the feasibility and effect of using the network platform to provide remote group interactive late pregnancy education to pregnant women.

No statistically significant difference was found between the intervention group and the control group in general characteristics (age, education level, employment status, etc.) and obstetric characteristics (number of pregnancies, age, etc.) (Tables 1, 2). These results showed that the distribution of each group was uniform. In addition, we also identified statistically significant differences before and after the intervention. Before the intervention, the scores of pregnancy stress and each dimension of pregnant women in the intervention and control groups were not statistically significant and were at a mild level (Table 3). After the routine intervention, the pregnancy stress and scores of all dimensions of pregnant women in the control group increased, but without statistical significance (*p* > 0.05). From the perspective of the Health Belief Model, such limited effectiveness is attributable to low perceived benefits of attending childbirth preparation courses and substantial perceived barriers—crowded facilities, time constraints, long travel distances, and urban traffic congestion (39). Work–family conflict, commuting distance, and limited healthcare access—instrumental support deficits as conceptualized by House’s Social Support Theory—constitute structural barriers that impede sustained engagement with in-person antenatal education (40). Consequently, expectant mothers are increasingly turning to digital platforms, which transcend spatiotemporal barriers to provide accessible and essential guidance on pregnancy, childbirth, and parenting (41). To alleviate the pressure of pregnancy and to meet the needs of pregnant women for maternal knowledge, the Digital-integrated health education mode highlights the unique advantages of health education compared with traditional routine health education. In this study, the pregnant women in the intervention group participated in the Digital-integrated GPNC. The scores of the three dimensions of pregnancy pressure score, maternal anxiety, maternal health, and worry about physical changes and other factors were significantly lower than before intervention (*p* < 0.05). This suggests that scientific and effective pregnancy health education was associated with positive outcomes in pregnant women, and may contribute to enhancing their self-efficacy, reduce negative emotions such as anxiety, depression, and fear during pregnancy, and alleviate the pregnancy-related pressure.

A previous survey study (42) showed that most pregnant women believe that the healthcare knowledge obtained from medical personnel during pregnancy is the most comprehensive and practical. Therefore, the perinatal distance education system in the information age should give full play to the guiding role of medical personnel so that pregnant women can obtain full and reasonable information support, confidence, and trust during the third trimester, highlighting the critical value of professional support provided by medical personnel. In the third trimester, the Digital-integrated GPNC is mainly controlled by midwives, and pregnant women who receive health education are added to an online group. Through the Internet social media, the midwives and the pregnant women in the group are more convenient and efficient, which is conducive to the development and monitoring of the professional support of medical staff. The study showed that before the intervention, there was no significant difference in the scores of pregnant women’s delivery expectation and each dimension between the intervention group and the control group (all *p* > 0.05). After the intervention, there were significant differences in the scores of pregnant women’ expectations of pregnancy and delivery and the dimensions of “medical environment,” “spouse support”, and “control and participation” between the two groups (all *p* < 0.05). Also, the scores of pregnant women in the intervention group were significantly higher than those in the control group. The scores of “spouse support”, “control and participation” and “medical support” of pregnant women in the intervention group were significantly higher than those before the intervention. Also, the dimension of “labor pain expectation” was lower than that before the intervention (*p* < 0.05), which indicates that the Digital-integrated GPNC in the third trimester of pregnancy was associated with providing pregnant women with more professional support, contributing to enhanced self-efficacy during pregnancy and improved childbirth expectancy. Compared with the regular education mode, the Digital-integrated GPNC in the third trimester of pregnancy emphasizes the importance of family participation. The family members of the group participants were regularly gathered through the network platform, and the video messages and webcasts were used to teach the points of maternal and child care. The pregnancy and childbirth information was made accessible to the group members making health-related knowledge free of charge. Because of the transmission and control of medical professional support, families could supervise pregnant women at any time in their daily life and help them timely change unhealthy behaviors, thus improving their health. At the same time, this helped avoid unnecessary family conflicts between husband and wife and family members caused by the lack of knowledge of pregnancy and childbirth. Positive and effective spouse and family support and emotional support in families are also conducive to alleviating the negative psychological experience related to pregnancy and childbirth in the later stage of pregnancy. The family-centered participatory health education meets the requirements of holistic nursing and the family breeding support model.

In order to ensure the homogeneity of the study and eliminate the interference of physical and mental factors of pregnant women on natural delivery, we compared the delivery of primiparas in natural delivery between the intervention group and the control group, finding significant differences in the two indexes of total labor process and the time of the first labor process between the two groups, where the time of the intervention group was shorter than that of the control group (*p* < 0.05). The evaluation of the delivery experience of primiparas in the two groups of natural delivery showed a significant difference in the score of “perceived environmental safety” between the two groups (*p* < 0.05), with the score of pregnant women in the intervention group being significantly higher than in the control group. Research shows that the use of active and effective pregnancy health education model for pregnant women was associated with improvements in their attitude toward pregnancy and delivery, eliminate the fear of delivery, improve the quality of uterine contraction, shorten the labor process, promote natural delivery and reduce the cesarean section rate (43, 44). The content, quality, and presentation of health education are the most important for health education during pregnancy. The development of the Internet information age also changed people’ learning habits and learning methods. Although social media is suitable for information dissemination, it may increase public fear and reduce social trust by providing false information. In order to meet the new needs of health education, an appropriate health education model is needed. Therefore, the pregnancy group distance education led by medical staff can ensure the content quality of pregnancy health education and flexible use of the network platform and media to provide pregnant women with pregnancy knowledge and information without time and space constraints (26, 45). In the face of the stress and pressure of the environment, the demand for social support is even more prominent.

In late pregnancy, the Digital-integrated GPNC enables pregnant women to release negative emotions, share their problems early in the discussion and obtain professional responses from the medical staff. The model also provides a safe space for sharing and confirming relevant information about pregnancy, as it has become the main source of information for pregnant families on admission preparation, delivery experience, and hospitalization experience. The model embodied the high score of the dimension of “perceived environmental safety” among pregnant women in the intervention group, which indicates the confidence of pregnant families in childbirth.

## 6 Advantages, limitations, and future research

The advantage of this study is that it confirmed the effectiveness of the Digital-integrated GPNC, which can overcome space and time-related limitations, improve the expectancy of childbirth, and reduce the stress caused by anxiety, fear of childbirth, and parenting problems, thus making it a valuable supplement to health education during pregnancy, enriching the form of health education during pregnancy. Under this mode, the professional, family, and peer support available in the same group form a strong social support system that can meet the diversified needs of pregnant women. Especially with the accelerating pace of modern life and the rapid development of digital networks, prenatal, postnatal, and postnatal-related knowledge and skills in online education and demonstration have improved the accessibility, effectiveness, and utilization of healthcare during pregnancy, thus improving the quality of obstetric healthcare services.

From a practical perspective, the Digital-integrated GPNC model can be integrated into routine prenatal care services through the following strategies: (1) embedding online group sessions within existing antenatal care schedules, with midwives trained in both clinical assessment and digital facilitation; (2) selecting accessible technology platforms that require minimal digital literacy, with technical support available for participants; (3) establishing quality control indicators including session attendance rates, participant satisfaction scores, and outcome monitoring metrics; and (4) developing tiered implementation models that accommodate varying levels of institutional resources and digital infrastructure.

The limitations of this study are as follows. First, due to the non-randomized, quasi-experimental design, participants self-selected into the intervention group, which may introduce selection bias. Women who voluntarily chose to participate in the Digital-integrated GPNC may have differed systematically from those receiving routine care in terms of motivation, health awareness, educational engagement, and digital literacy. Although we matched participants by gestational week and age, and baseline comparisons confirmed no significant differences in general and obstetric characteristics (Tables 1, 2), residual confounding from unmeasured factors cannot be entirely excluded. Due to the non-randomized design, causal inferences cannot be drawn, and the observed associations may be influenced by residual confounding factors. Second, this study was conducted at a single tertiary hospital in an urban area of Chongqing, China. The findings may not be generalizable to other healthcare systems, rural areas with limited digital infrastructure, or populations with different levels of digital literacy or access to technology. The digital divide may prevent certain populations from benefiting from this model, potentially exacerbating health inequities. Third, all psychological outcomes were assessed through self-reported questionnaires (PPS, CEQ, CEQ-C), which may introduce recall bias and social desirability bias. Future studies could incorporate objective physiological measures to complement self-reported data. Fourth, although Analysis of Covariance (ANCOVA) is recommended for non-randomized pre-post designs, the non-normal distribution of our data precluded its valid application. While baseline comparability and within-group pre-post analyses partially address this concern, future studies with larger sample sizes should consider using generalized estimating equations (GEE) or mixed linear models. Finally, since distance education requires professional equipment such as Internet access and smartphones, the research results cannot be extended to people without such equipment or limited Internet access.

Since the Digital-integrated GPNC is family-centered, participatory health education, spouses and other family members can participate online. Therefore, future research should adopt qualitative methods to interview couples and explore different psychological experiences and feelings. The sample size should also be further expanded to explore whether the education model impacts the choice of delivery mode and delivery outcome of primipara. Multi-center studies across diverse geographic and socioeconomic settings are needed to validate the generalizability of these findings. From a policy and practice perspective, future efforts should focus on: (1) developing standardized operating procedures (SOPs) for implementing Digital-integrated GPNC within existing prenatal care pathways, including protocols for session structure, midwife training, and quality assurance indicators; (2) addressing the digital divide by providing alternative access modalities (e.g., telephone-based support, community digital hubs) for populations with limited digital literacy or technology access, to ensure that the model does not exacerbate health inequities; (3) training healthcare personnel in digital health competencies, including online group facilitation skills and remote monitoring techniques; and (4) establishing sustainable funding models and reimbursement mechanisms to support the integration of synchronous digital care into routine maternal health services.

## Data Availability

The original contributions presented in the study are included in the article/supplementary material, further inquiries can be directed to the corresponding authors.
